# Autoencoder-Enhanced Hierarchical Mondrian Anonymization via Latent Representations

**DOI:** 10.3390/e28040372

**Published:** 2026-03-25

**Authors:** Junpeng Hu, Tao Hu, Zhenwu Xu, Jinan Shen, Minghui Zheng

**Affiliations:** 1 School of Cyber Science and Engineering, Sichuan University, Chengdu 610207, China; 2021326240017@stu.scu.edu.cn (J.H.); xuzhenwu_xzw@163.com (Z.X.); 2 College of Intelligent Science and Engineering, Hubei University for Nationalities, Enshi 445000, China; 16671054751@163.com (T.H.); shenjinan@hbmzu.edu.cn (J.S.)

**Keywords:** data anonymization, k-anonymity, autoencoder, latent space partitioning, microaggregation, linkage-attack risk

## Abstract

Releasing structured microdata requires balancing utility and privacy under group-based disclosure risks. We propose AE-LRHMA, a hybrid anonymization framework that performs Mondrian-style hierarchical partitioning in an autoencoder-learned latent space and integrates local (*k*,*e*)-microaggregation. To explicitly control sensitive-value concentration and diversity within each equivalence class, we introduce a tunable constraint set consisting of *k*, a maximum sensitive proportion threshold, and an optional sensitive-entropy threshold (used as a hard gate when enabled and otherwise as a soft term in split scoring). The anonymized output is generated via standard interval/set generalization in the original space. Experiments on Adult and Bank Marketing demonstrate that AE-LRHMA yields lower information loss and more stable group structures than representative baselines under comparable settings. We further report linkage-attack-oriented risk metrics to empirically characterize relative disclosure trends without claiming formal guarantees, such as differential privacy.

## 1. Introduction

With the increasing sharing and reuse of structured microdata in domains such as finance, healthcare, and education, data publication must balance regulatory compliance with analytical utility. A typical released table contains quasi-identifiers (QIs) and sensitive attributes (SAs). When an attacker possesses auxiliary information and can match records on QIs, linkage attacks may re-identify individuals or infer sensitive values, resulting in privacy leakage. Therefore, practical data publishing requires anonymization methods that provide an interpretable and adjustable privacy–utility trade-off under realistic linkage-attack assumptions [1,2].

For structured tabular data, k-anonymity and its extensions remain mainstream equivalence class (EC)-based anonymization frameworks [1]. Representative methods, such as Mondrian and MDAV, construct ECs through partitioning or grouping and apply interval/set generalization to QIs, thereby reducing re-identification risk under standard linkage assumptions [3,4,5]. Extensions such as ℓ-diversity and t-closeness further constrain sensitive value distributions within ECs and are intended to mitigate attribute-inference risks that cannot be sufficiently controlled by EC size alone [6,7].

However, when the QIs are high-dimensional, strongly correlated, and partially nonlinear, existing EC-based anonymization approaches still face three practical challenges. First, partitioning or distance measurement performed directly in the original QI space often fails to capture nonlinear correlation structures, which may cause metric distortion, fragmented ECs, and unstable information loss [4,8]. Second, sensitive distribution constraints are often enforced in a coarse or post hoc manner, providing limited process-level control over worst-case situations, such as excessive concentration of a sensitive value or overly low within-class diversity [9,10]. Third, although representation learning has been explored in privacy-related studies, its workflow-level integration with interpretable k-anonymity family mechanisms remains insufficient, especially when the EC geometry and sensitive distribution need to be controlled within a unified release framework [11,12,13,14,15].

To address these challenges, we propose AE-LRHMA, an autoencoder-enhanced hierarchical Mondrian anonymization method in the latent space. The method first learns a low-dimensional latent space for the QIs to regularize correlation structures. It then performs Mondrian-style hierarchical partitioning in that latent space. To move beyond the conventional “split first, repair later” workflow, AE-LRHMA incorporates the EC size and sensitive distribution constraints directly into split feasibility checking and split scoring. A local (*k*,*e*)-microaggregation procedure is further used to stabilize the within-group structure, after which the resulting partitions are mapped back to the original space for standard interval/set generalization. For evaluation, we use the normalized certainty penalty (NCP) to measure utility loss and to adopt linkage-attack-oriented indicators, such as the ERR and UMR, to characterize empirical disclosure-risk trends.

**Figure 1:**
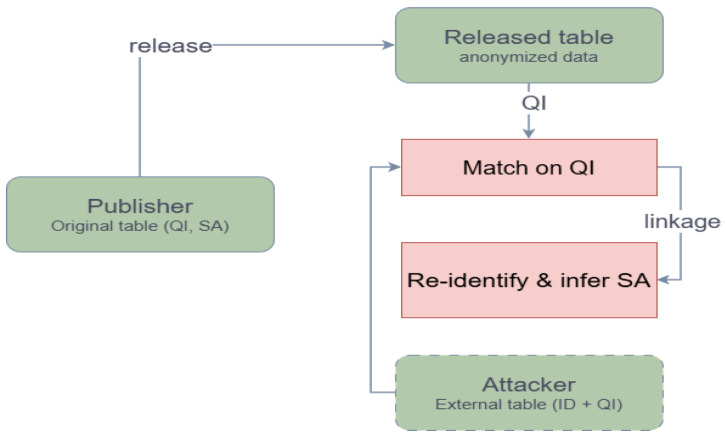
Publishing–linkage-attack threat model.

This paper focuses on a one-time release scenario under a publishing–linkage-attack threat model. In this setting, a publisher releases an anonymized table containing generalized QIs, while an attacker may hold an auxiliary table with explicit identifiers and overlapping QIs and attempt a record linkage. Our goal is to achieve a more stable and controllable privacy–utility trade-off under this release setting, rather than to provide formal guarantees in the sense of differential privacy. Instead, the proposed hybrid model provides interpretable, group-based controls on disclosure risk under the considered publishing–linkage-attack setting. Figure 1 illustrates this threat model.

The main contributions of this paper are summarized as follows:

1. **An autoencoder-enhanced hierarchical framework for structured microdata anonymization.** We propose AE-LRHMA, an end-to-end anonymization framework for the one-time release of static structured data that combines autoencoder-based latent representations, hierarchical Mondrian partitioning, and local microaggregation.
2. **Latent space partitioning with process-level sensitive distribution control.** To improve anonymization stability under high-dimensional and correlated QIs, we perform hierarchical partitioning in a learned latent space and directly embed EC size and sensitive distribution constraints into split feasibility checking and split scoring, rather than relying on post hoc repair.
3. **An interpretable hybrid privacy control mechanism within an EC-based workflow.** We use explicit and tunable constraints, including maximum sensitive proportion and entropy-related control, together with local (*k*,*e*)-microaggregation, to provide an interpretable risk control mechanism from partitioning to final generalization output.
4. **Systematic evaluation under the target release-and-linkage setting.** On the Adult and Bank Marketing datasets, we evaluate the proposed method in terms of utility loss (NCP), efficiency, and attacker-oriented empirical risk indicators (ERR/UMR), showing that AE-LRHMA achieves a more balanced privacy–utility trade-off than the compared methods under the considered threat model.

## 2. Related Work and Background

### 2.1. Equivalence Class Anonymization Models and Sensitive Distribution Constraints

For the one-time release of structured tabular data, equivalence class (EC) anonymization remains one of the most widely adopted paradigms. k-anonymity partitions record into ECs and generalize quasi-identifiers (QIs) so that each record becomes indistinguishable from at least *k* − 1 others within its EC, thereby reducing re-identification risk under a typical publishing–linkage-attack assumption [1]. In practice, representative methods, such as Mondrian and MDAV, follow a relatively stable implementation workflow of “partitioning/aggregation + generalization output”, which facilitates deployment in real-world anonymization pipelines [16,17].

However, k-anonymity mainly constrains EC size and does not directly regulate the potential homogeneity of sensitive attributes within an EC; thus, attribute inference may still occur. To mitigate this limitation, ℓ-diversity and t-closeness incorporate constraints on the sensitive attribute (SA) distributions: ℓ-diversity emphasizes diversity of sensitive values within each EC, while t-closeness limits the deviation of an EC’s SA distribution from the global distribution, thereby reducing the worst-case concentration of sensitive values [6,7].

Although these models bring SA distributions into the constraint set, many implementations still treat the sensitive distribution conditions as an end-stage check or a coarse-grained gate [18,19]. This makes it difficult to finely control worst-case situations inside ECs, such as an excessively high proportion of a sensitive value or overly low sensitive entropy [20,21]. This reveals a gap between distribution-aware privacy models and process-level controllability in practical anonymization workflows. Under a publishing–linkage-attack setting, this limitation motivates approaches that move sensitive distribution control from post hoc inspection toward earlier decision stages in the anonymization workflow.

### 2.2. Partition-Based Anonymization for High-Dimensional Correlated Data: Mondrian Variants and Limitations

Among EC-based anonymization algorithms, Mondrian represents a canonical paradigm of “recursive binary partitioning + leaf generalization release”. It repeatedly selects a split dimension and a cut point to divide a record set into subsets until size or stopping conditions are met, and then applies interval/set generalization to the QIs at leaf nodes. This workflow is intuitive, interpretable, and easy to implement, and it can be extended to accommodate more complex privacy constraints; hence, Mondrian and its variants have been widely used and continuously improved in both research and practice.

The existing research around Mondrian has mainly progressed along the following three directions: (i) extending Mondrian to satisfy more sophisticated privacy models and constraints while preserving usability; (ii) developing distributed or parallel implementations for large-scale processing; and (iii) improving partition–structure quality by introducing structured mappings or stronger organization mechanisms to stabilize partition boundaries and EC shapes [4,8,9].

Nevertheless, when the QIs are high-dimensional, strongly correlated, and exhibit nonlinear relationships, axis-aligned splitting in the original attribute space becomes prone to metric distortion and fragmentation. Heuristics based on spans or local distances may fail to capture nonlinear correlation structures, leading to irregular and overly fragmented ECs and making information loss harder to control in a stable manner [4,8]. Accordingly, an important research direction is to retain Mondrian’s interpretable hierarchical grouping backbone while improving partition stability in complex, correlated data settings.

### 2.3. Microaggregation and Grouping Stability: MDAV/(k,e)-MDAV and Partition–Aggregation Synergy

Different from Mondrian’s partition-based generalization route, MDAV exemplifies another important line—microaggregation—which groups similar records and applies representative or interval-based transformations to satisfy the group size constraints while reducing the information loss. For large-scale data, MDAV and its variants, including accelerated versions for big data, have evolved toward better efficiency and scalability, forming a relatively mature aggregation-based anonymization family [3,4,5].

From a publishing–linkage-attack perspective, however, microaggregation may encounter practical tensions under complex distributions. When distributions are heterogeneous, constraints are numerous, or local density is uneven, aggregation can produce boundary residual samples and unstable small clusters, making the overall EC structure difficult to control [22,23]. Accordingly, a more robust trend in research and practice is to combine hierarchical partitioning with local microaggregation: hierarchical partitioning provides an interpretable grouping framework, while local aggregation enhances within-group stability and alleviates boundary effects.

Moreover, as feature sources become more complex, multi-view collaborative microaggregation has been used to accommodate multi-source and multi-view feature organizations, better exploiting complementary information and maintaining grouping stability [24]. This suggests that, when the goal goes beyond merely meeting the group size constraints and also requires structure preservation and multi-constraint coordination, partition–aggregation synergy is often more suitable than a single aggregation strategy alone.

### 2.4. Latent Representation-Driven Anonymization: Representation Learning and Integration with the k-Anonymity Workflow

In recent years, the intersection of representation learning and privacy protection has attracted increasing attention. Particularly in high-sensitivity domains such as healthcare, surveys and empirical studies emphasize that real-world data sharing requires balancing utility and risk control, and they summarize the trade-offs of different anonymization strategies in practice [11,12,13]. In parallel, risk evaluation studies under publishing–linkage attacks have gradually developed operational indicator systems, enabling a comparison of the relative risk trends across anonymization strategies under a unified threat assumption [25,26,27].

Along the direction of combining representation learning with EC-based anonymization, prior work has attempted to learn structured mappings or low-dimensional organization mechanisms to improve the stability of the original space partitioning. Overall, however, the workflow-level and interpretable integration of deep representation learning with hierarchical partitioning in the k-anonymity family remains limited, and systematic solutions that can simultaneously control EC geometry and sensitive distributions within a unified framework are still lacking [14,15].

From a procedural viewpoint, traditional hierarchical partitioning anonymization often follows the pattern of “split by geometric heuristics, check constraints, and patch if necessary”. This paradigm has the following two typical drawbacks: (i) splitting decisions are mainly geometry-driven and may produce ECs that satisfy size constraints but severely violate sensitive distribution balance; and (ii) post hoc patching introduces extra perturbations and may amplify information loss, making the privacy–utility trade-off less stable. These limitations motivate approaches that incorporate process-level constraint-driven control into hierarchical partitioning while leveraging learned latent representations to improve grouping stability in high-dimensional correlated scenarios. The present study is positioned along this line.

## 3. Preliminaries

### 3.1. Problem Setting and Threat Model

We focus on a one-time release scenario of static structured (tabular) data. As illustrated in Figure 1, a data publisher anonymizes an original dataset containing quasi-identifiers (QIs) and sensitive attributes (SAs) and then releases the anonymized table. Meanwhile, an attacker may possess an external auxiliary table that includes explicit identifiers and overlaps with the released table on the QI fields. By matching records on the QIs, the attacker can perform record linkage to re-identify individuals and infer their sensitive values. Our objective under this publishing–linkage-attack setting is to achieve a more stable and controllable privacy–utility trade-off and a clear risk reduction trend, rather than providing formal guarantees in the sense of differential privacy.

Under this setting, anonymization is typically implemented by interval/set generalization on the QIs so that records sharing the same generalized QI representation form an equivalence class (EC), thereby reducing individual distinguishability in the QI space. For any given generalization scheme, the dataset can be partitioned into multiple ECs, where each EC consists of the records having identical generalized QI values. The classical k-anonymity model requires that the size of every EC be at least *k*, which can reduce re-identification risk under certain assumptions in linkage attacks. However, constraining the EC size alone is insufficient to regulate the attribute inference risk caused by sensitive value homogeneity within an EC. Therefore, it is necessary to additionally control whether the sensitive distribution inside an EC is overly concentrated or sufficiently diverse.

In summary, under the publishing–linkage-attack setting, the EC size constraint *k* alone cannot effectively suppress sensitive value homogeneity within the ECs and may still lead to attribute inference. To make risk control operational, we adopt sensitive distribution constraints (as introduced in Section 2.1) and explicitly treat them as *process-level* criteria and optimization objectives during subsequent splitting and within-group adjustment, enabling an interpretable and tunable risk reduction behavior.

### 3.2. Basic Workflow and Limitations of Mondrian Hierarchical Partitioning

Mondrian-style approaches represent a typical hierarchical partitioning-based generalization paradigm in EC anonymization. They recursively construct a binary partition tree: at each level, a split dimension and a cut point are selected to divide a record set into two subsets until the size and stopping conditions are satisfied; then, interval/set generalization is applied to leaf nodes to produce the released data. This workflow is intuitive, interpretable, easy to implement, and can be extended to support more complex constraints; hence, it has been widely adopted in both research and engineering practice [4,8,9].

However, when the QIs are high-dimensional, strongly correlated, and exhibit nonlinear dependence, directly performing axis-aligned splitting in the original QI space (or describing similarity using common distance metrics) often fails to reflect the latent structure accurately. On the one hand, metric distortion may occur; on the other hand, partition boundaries can become unstable and the ECs may become fragmented, which in turn leads to fluctuating information loss and imbalanced sensitive distributions [4,8]. Therefore, while preserving the interpretability of hierarchical partitioning, improving the stability of partition structures and reducing fragmentation is a fundamental issue that the subsequent method design must address.

### 3.3. Microaggregation and an Intuitive View of (k,e)-MDAV

Microaggregation methods group similar records and apply representative/interval-based transformations to satisfy the group size constraints while minimizing information loss. For large-scale data, MDAV and its variants have been extended toward improved efficiency and scalability [3,5]. Moreover, multi-view collaborative microaggregation can adapt to multi-source and multi-view feature organization, demonstrating the potential of microaggregation to handle complex data structures [24]. In scenarios with complex distributions and multiple constraints, relying solely on “partitioning” or solely on “aggregation” is often insufficient to simultaneously maintain interpretability and within-group stability. A more robust strategy is to combine hierarchical partitioning + local microaggregation: hierarchical partitioning provides an interpretable grouping backbone, while local microaggregation enhances within-group similarity and alleviates boundary effects as well as small-cluster/residual-sample issues caused by uneven density, thus producing more stable final EC structures.

In our framework, *k* corresponds to the minimum EC size requirement. The parameter *e* serves as a local constraint in the microaggregation stage of (*k*,*e*)-MDAV, restricting the spread of sensitive dissimilarity during local aggregation so that sensitive risk control remains interpretable while grouping is stabilized. Importantly, *e* does not change the main mechanism of our approach (latent space hierarchical partitioning and constraint-aware splitting); instead, it acts as a local refinement component that works collaboratively with them to improve the stability of the overall privacy–utility trade-off.

### 3.4. Utility and Risk Metrics

**Figure 2:**
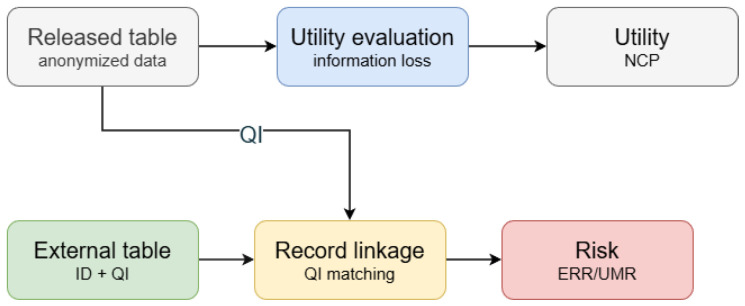
Utility–risk evaluation pipeline under linkage attacks.

To compare the privacy–utility trade-offs of different anonymization strategies and parameter settings under a unified framework, we evaluate both utility and risk (Figure 2). On the utility side, we use the NCP (normalized certainty penalty) to quantify the information loss caused by QI generalization. For numerical attributes, the NCP measures the generalization strength via the EC interval length relative to the global range; for categorical attributes, it measures ambiguity via the EC value set size relative to the total number of categories. The overall information loss is computed as a weighted aggregation of the attribute-wise NCP, which directly reflects the degradation of analytical precision in the released data.

On the risk side, we adopt an operational evaluation pipeline from the publishing–linkage-attack perspective. The attacker performs record linkage by matching the QI fields between the external auxiliary table and the released table, and empirical risk indicators are computed to characterize trends in re-identification and attribute inference risks [25,26,27]. Specifically, we use containment linkage to construct candidate EC sets: if the QI values of an external record are contained by the generalized interval/set of an EC in the released table for every QI, then this EC is considered a candidate. A smaller candidate set implies a smaller attacker search space; when the candidate set size equals 1, the external record can be uniquely localized to a single EC.

Based on the candidate sets, we use two complementary empirical risk metrics. The UMR (unique match rate) measures the proportion of external records that can be uniquely mapped to exactly one candidate EC. The ERR (expected re-identification risk) estimates an upper bound of the identity hit risk under a “most favorable candidate” strategy: the attacker prioritizes the smallest EC in the candidate set and uniformly guesses an individual within that EC, yielding the expected hit rate. We emphasize that the ERR/UMR are empirical risk measures for comparative analysis under a specified threat model and are not equivalent to formal privacy guarantees such as differential privacy. In this paper, they are used together with the NCP to form a two-sided utility–risk evidence chain, enabling a clearer presentation of the trade-offs across the anonymization strategies and parameter settings (Figure 2).

## 4. AE-LRHMA Anonymization Algorithm

### 4.1. Design Rationale and Overall Framework

**Figure 3:**
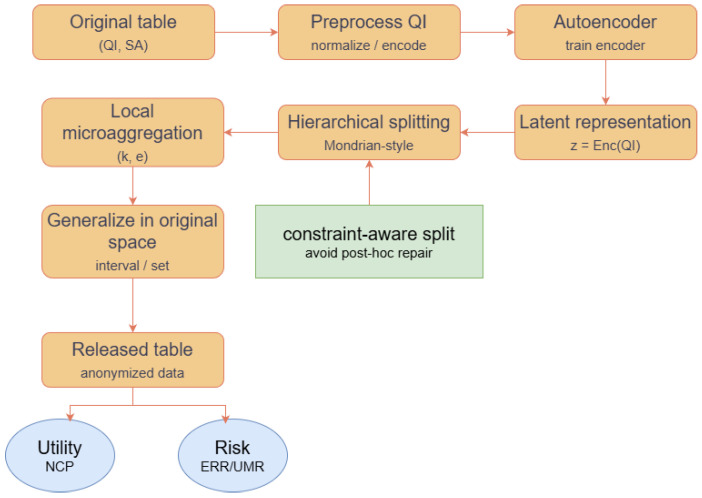
Overall workflow of AE-LRHMA.

Under the publishing–linkage-attack threat model, we propose an autoencoder-enhanced hierarchical Mondrian anonymization method, termed AE-LRHMA. The key idea is to use latent representations as a bridge to unify the workflow of data preprocessing → representation learning → hierarchical partitioning → generalization release, and to introduce a constraint-aware splitting mechanism during partitioning to avoid the instability caused by the conventional “split first, repair later” paradigm. The overall framework and data flow among the modules are illustrated in Figure 3.

Within this framework, we further define a hybrid privacy constraint that jointly characterizes both the equivalence class (EC) size and the sensitive attribute (SA) distribution, providing explicit criteria for constraint-aware splitting and subsequent local microaggregation. Let the original dataset be $D=\left\{\left.\left(q_{i},s_{i}\right)\right|i=1,\cdots ,n\right\}$, where $q_{i}\in R^{d}$ denotes the quasi-identifier (QI) vector of record *i* (e.g., age, occupation, education, and contact type), and $s_{i}$ denotes the corresponding sensitive attribute (SA) value. Let Q be the set of QI attributes and S be the set of SA attributes. Given a generalization scheme, records with the same generalized QI values form an equivalence class (EC) G⊆D. Then, denote the EC partition of D as $G=\left\{G_{1},\cdots ,G_{m}\right\}$. A consolidated list of symbols and notations is provided in Supplementary Table S3.

To jointly characterize the EC size and SA distribution, we introduce two indicators on top of the classical k-anonymity model: the maximum sensitive proportion and the sensitive entropy. For a given EC G, the empirical distribution of SA values is defined as follows:

(1) $$P_{G}\left(v\right)=\frac{\left|\left\{\left(q_{i},s_{i}\right)\in \left.G\right|s_{i}=v\right\}\right|}{\left|G\right|}$$

Based on the above distribution, the maximum sensitive proportion and the sensitive entropy are respectively defined as follows:

(2) $$p_{\max\left(G\right)}=\underset{v}{max}P_{G}\left(\nu \right)$$

(3) $$H\left(G\right)=-\sum_{v}P_{G}\left(v\right)\log P_{G}\left(v\right)$$

Here, $p_{max}\left(G\right)$ reflects the concentration of a certain sensitive value within an EC, while $H\left(G\right)$ reflects the diversity of sensitive values. A higher $p_{max}\left(G\right)$ indicates a higher risk of attribute inference, and a lower entropy indicates weaker diversity. We therefore adopt the following hybrid privacy constraints to restrict the EC size and sensitive distribution simultaneously:

(4) $$\left|G\right|\geq k\;,p_{max}\left(G\right)\leq p_{max}\;,\;H\left(G\right)\geq h_{min}$$

In addition, a microaggregation is integrated into the framework to further stabilize utility and provide finer-grained control over the EC characteristics. Specifically, if there exists an EC partition G, such that every G∈G satisfies the constraints in Equation (4), and the sensitive attribute dissimilarity during local microaggregation does not exceed a threshold e, then the dataset D is said to satisfy $\left(k,e,p_{max},h_{min}\right)$-anonymity. Compared with the traditional k-anonymity and ℓ-diversity models, this definition retains the EC size constraint while explicitly controlling both the concentration and diversity of sensitive information within each EC via $p_{max}$ and an optional entropy hard-gate threshold $h_{min}$, thereby offering a more fine-grained “knob” at the group privacy level.

From a privacy-interpretation perspective, the proposed hybrid model should be understood as providing group-based and threat model-dependent controls under one-time release. Specifically, *k* constrains identity ambiguity through minimum EC size, $p_{max}$ limits the concentration of the most frequent sensitive value within an EC, and *H*(*G*)/$h_{min}$ constrain the within-EC diversity. The parameter *e* further acts as a local stabilizer during microaggregation. These controls are not formal probabilistic guarantees in the sense of differential privacy, nor are they exact models of an attacker’s true inference probability; instead, they serve as interpretable operational controls on identity ambiguity, sensitive value concentration, and within-EC diversity under the publishing–linkage-attack setting.

AE-LRHMA combines autoencoder-based latent representations, hierarchical Mondrian-style partitioning, local (*k*,*e*)-MDAV, and sensitive distribution constraints to balance privacy protection and data utility in high-dimensional settings [4]. Compared with original space partitioning, these latent representations provide a more regular basis for grouping and generalization. The detailed workflow, including preprocessing, representation learning, partitioning, and generalization output, is described in Section 4.3.

### 4.2. Constraint-Aware Splitting Mechanism

Traditional hierarchical partitioning anonymization often follows a “split by span, check constraints, and patch if needed” workflow, which leads to two issues. First, splitting directions are mainly driven by geometric structure and can easily produce ECs that meet size requirements but exhibit excessive concentration in sensitive distributions. Second, post hoc patching introduces additional perturbations and amplifies information loss. To address this, AE-LRHMA moves constraint checking into every split decision, shifting the process from “post hoc patching” to “constraint-driven” splitting.

Concretely, for a current node corresponding to a candidate EC, we generate multiple split candidates in the latent space. For each candidate split producing left and right subsets, AE-LRHMA jointly evaluates the following:

1. Size constraint (k): if any subset has fewer than k records, the split is infeasible.
2. Upper bound on sensitive concentration ($p_{max}$): if the maximum proportion of any sensitive value in a subset exceeds $p_{max}$, the split is infeasible or receives a strong penalty in scoring.
3. Entropy-based diversity control ($h_{min}$, optional hard gate): if a subset’s sensitive entropy is below $h_{min}$, the split is infeasible or receives a strong penalty; when $h_{min}$ = 0, we disable the hard entropy gate but still encourage entropy-balanced splits through the entropy-improvement term in the composite split-scoring function (Equation (7)).

Among feasible candidates, AE-LRHMA further leverages the latent space structural information to select a splitting direction that yields lower information loss and is more favorable for subsequent microaggregation. In this way, k, $p_{max}$, and $h_{min}$ are not merely “final constraints” but explicit control variables that directly participate in split decisions, explaining their controllable and tunable role in the privacy risk adjustment.

In addition, the parameter e constrains local sensitive dissimilarity in the (*k*,*e*)-MDAV microaggregation stage, limiting the spread of sensitive differences during local aggregation. Notably, e does not change the main contribution (latent space hierarchical partitioning with constraint-aware splitting) but works collaboratively to improve the overall privacy–utility balance.

### 4.3. Algorithm Workflow and Key Modules

Let the input dataset be D, where $q_{i}$ is the QI vector and $s_{i}$ is the SA value. Key hyperparameters include the following: k-anonymity parameter k, local sensitive-dissimilarity threshold e, maximum sensitive proportion threshold $p_{max}$, sensitive optional entropy hard-gate threshold $h_{min}$, maximum group size max_group_size, latent dimension r, autoencoder training epochs, and weight coefficients in the composite split-scoring function.

(1) Data preprocessing.

First, determine the QI set and SA set and extract the corresponding fields. For numerical QIs, perform missing-value imputation, outlier clipping, and z-score normalization. For categorical QIs, we map categories into numeric codes to obtain fixed-dimensional input vectors for the AE (integer encoding in our experiments). We note that integer encoding may introduce an artificial ordinal relationship; thus, we discuss this choice as a limitation. After preprocessing, all QI features form a matrix X, where each row represents a record in a d-dimensional QI feature space.

(2) Autoencoder-based representation learning.

An autoencoder composed of an encoder and a decoder is trained in an unsupervised manner with X as input. The encoder maps the input from d dimensions to an r-dimensional latent space through stacked fully connected layers with nonlinear activations; the decoder uses a roughly symmetric structure to reconstruct inputs back to the original dimension. Parameters are optimized by minimizing the mean reconstruction loss as follows:

(5) $$L\left(\theta ,\phi \right)=\frac{1}{n}\sum_{n=1}^{n}{\left\|x_{i}-g_{\phi }\left(f_{\theta }\left(x_{i}\right)\right)\right\|}_{2}^{2}$$

Regularization and dropout may be used to improve generalization. After training converges, only the encoder is kept to compute latent representations for all samples, as follows:

(6) $$Z=f_{\theta }\left(X_{Q}\right)\in R^{n\times r}$$

where the i-th row vector is the latent representation of record i.

(3) Latent space Mondrian partitioning and composite scoring.

Using the latent matrix as the input, the algorithm recursively performs Mondrian-style binary partitioning in the latent space. For a current node, if its size is below 2k or it reaches the maximum group size max_group_size, it is marked as a leaf and no further split is performed. Otherwise, the algorithm computes the per-dimension statistics (min, max, span), selects the dimension with the largest span as the split axis, and splits the node at the median (or a quantile) into left and right subsets. It then computes the sensitive distribution statistics on both sides, including $p_{max}$ and sensitive entropy H. To preserve structural information while controlling sensitive distributions, a composite split-scoring function is constructed as follows:

(7) $$Score\left(G\to G_{L},G_{R}\right)=\alpha \cdot \Delta_{span}+\left(1-\alpha \right)\cdot \Delta_{H}$$

Here, one term measures the weighted change in the latent space span before/after splitting, another term reflects the entropy improvement on both sides relative to the parent, and the weights are tunable coefficients. A split is accepted only if both subsets satisfy feasibility checks (size constraint and threshold-based sensitive distribution constraints), and the composite score exceeds a preset threshold; otherwise, the current node becomes a leaf. This yields candidate ECs that satisfy size constraints while accounting for sensitive distributions.

(4) Local (*k*,*e*)-MDAV microaggregation and generalization output.

Although latent space partitioning outlines the EC structures, boundary effects and non-uniform distributions may still produce small clusters or leftover records that violate local constraints. Therefore, within each candidate leaf node, AE-LRHMA invokes a Local_LR_KEMDAV subroutine to perform local microaggregation following the (*k*,*e*)-MDAV principle: it ensures that each final EC has at least k records, and uses distance constraints to control within-group similarity and sensitive dissimilarity. The remaining records are assigned to the nearest valid EC based on a combined distance in latent space and sensitive space. Finally, the ECs are mapped back to the original QI space: numerical attributes are generalized to intervals, categorical attributes to value sets, while sensitive attributes remain unchanged to support downstream analysis and modeling. Merging all the ECs yields a published dataset satisfying $\left(k,e,p_{max},h_{min}\right)$-anonymity.

### 4.4. Risk Upper-Bound Estimation Under Linkage Attacks

To avoid restricting privacy claims to empirical metrics, we provide an interpretable upper-bound estimate of re-identification risk under a given external-knowledge setting and attack model. This attacker-side characterization complements the group-level interpretation of the hybrid constraints introduced in Section 4.1. Suppose an attacker holds an external table T drawn from the same distribution and knows the quasi-identifier set Q used by the released data. The attacker then performs containment linkage: for any external record *x*, if for every q∈Q, the value $x\left[q\right]$ is contained in the generalized interval/set of attribute q in some equivalence class $EC_{j}$ of the released table *D* then $EC_{j}$ is regarded as a candidate. This yields the following candidate equivalence class set:

(8) $$C\left(x\right)=\left\{E\left.C_{j}\right|x\left[Q\right]\;is\;contained\;in\;EC_{j}\left[Q\right]\right\}.$$

The candidate set size reflects the attacker’s localization difficulty: smaller candidate sets imply easier localization, while a singleton candidate set means unique localization to one EC. Note that EC localization is not equivalent to direct individual identification; without additional priors, identity uncertainty remains primarily constrained by EC size. Consider the attacker’s most favorable strategy: selecting the smallest EC among candidates, which is defined as follows:

(9) $$m\left(x\right)≜{min}_{EC\in C\left(x\right)}\left|EC\right|$$

When the candidate set is non-empty, under the assumption of “no extra priors and uniform guessing within an EC”, admits a proxy upper bound under the following stated assumption:

(10) $$P_{r}\left(hi\left.t\right|x\right)\leq \frac{1}{m\left(x\right)}$$

If the candidate set is empty, the record cannot be matched to any EC and its contribution is treated as zero. Taking expectation over all external records yields an upper-bound estimate of the overall re-identification risk as follows:

(11) $$ERR≜E_{x\sim T}\left[\frac{1}{m\left(x\right)}\right]$$

This indicator captures the expected conservative estimate of the identity hit risk under containment linkage and the most favorable candidate strategy; smaller values indicate a lower overall re-identification risk. In addition to the expected risk, we also consider the probability of being uniquely localized to an EC [25,26,27], defined as the unique match rate (UMR) as follows:

(12) $$UMR≜P_{r}\left(\left|C\left(x\right)\right|=1\right)$$

The UMR measures how frequently external records are uniquely localized to an EC via containment linkage. Importantly, the UMR and the expected risk bound focus on different aspects: the UMR measures the uniqueness of the EC localization, while the expected risk bound measures the expected proxy upper bound of identity hits after localization under the most favorable candidate. They may not move in the same direction (e.g., the UMR can be high while the expected risk bound remains low if the uniquely matched EC is still large). We emphasize that these are upper-bound estimates under stated assumptions rather than reproductions of the attacker’s true success rate [25,26,27].

These bounds correspond directly to the publishing constraints. *k*-anonymity ensures that each EC contains at least *k* records; even if an attacker achieves unique localization, identity uncertainty remains among at least *k* individuals, limiting success probability by roughly 1/*k* under uniform guessing. The sensitive distribution constraints $p_{max}$ and *H*(*G*)/$h_{min}$ control the concentration and diversity of sensitive values within the ECs: the best single-point sensitive guess is bounded by $p_{max}$, while increasing entropy-related control discourages highly homogeneous sensitive distributions and suppresses the worst-case inference risk. In this sense, the hybrid constraints should be interpreted as EC-level operational controls on the worst-case concentration and diversity, rather than exact probabilistic models of attribute-inference success, whereas the ERR and UMR characterize how these controls translate into the attacker-side risk under the stated linkage assumptions [25,26,27]. Section 5.8 further provides empirical validation of these trends.

### 4.5. Pseudocode and Complexity Analysis

The full pseudocode of AE-LRHMA is provided in Supplementary Table S6 (Algorithm S1). The algorithm takes as input the original dataset, QI set, SA set, privacy parameters *k*,*e*,$\;p_{max}$, $h_{min}$, autoencoder parameters, and related thresholds, and outputs an anonymized dataset. It first preprocesses the QIs and trains the autoencoder to obtain low-dimensional latent representations; then performs Mondrian-style recursive partitioning in the latent space, maintaining a queue of subsets to process; it uses sensitive dissimilarity and the composite constraint function Check_Constraints to select candidate subsets and a remainder set; next, it calls Local_LR_KEMDAV within each candidate subset to complete (*k*,*e*)-MDAV-based local grouping; finally, the remaining records are assigned to the nearest EC, and interval/set generalization is performed in the original QI space to produce the final anonymized dataset [5].

From a complexity perspective, let the original QI dimension be represented by *d*, let the latent dimension be *r*, let the number of records be *n*, and let the number of training epochs be *E*. In the representation learning stage, the dominant cost per epoch is approximately linear in *n* and *d*, giving a time complexity on the order of the following:

(13) $$O\left(E\cdot n\cdot d\right)$$

In the latent space hierarchical partitioning stage, each split requires sorting and scanning samples along a chosen dimension. Assuming the partition tree height is on the order of logn, the total time complexity for the entire partitioning tree is approximately as follows:

(14) $$O\left(r\cdot n\log n\right)$$

In the local (*k*,*e*)-MDAV microaggregation stage, if the final EC sizes are roughly on the order of k, the total aggregation overhead can be estimated accordingly. Overall, the total time complexity of AE-LRHMA can be approximated as follows:

(15) $$O\left(E\cdot n\cdot d+r\cdot n\log n+n\cdot k\right)$$

In practical settings, r is typically a small constant relative to d, so the algorithm scales well.

For space complexity, the algorithm needs to store the original QI matrix X, the latent representation matrix Z, the hierarchical partition tree, and the EC partition results. The total space cost is approximately as follows:

(16) $$O\left(n\cdot \left(d+r\right)\right)$$

For the dataset scales used in our experiments, this memory footprint is easily manageable on standard server configurations and can support larger-scale structured data anonymization.

## 5. Algorithm Performance Comparison Experiments

### 5.1. Datasets and Preprocessing

In this study, we use two public datasets—UCI Bank Marketing and Adult Census Income—as experimental benchmarks. Both contain typical demographic and behavioral features, making them suitable for evaluating the privacy–utility trade-off of tabular data anonymization methods [28,29]. Supplementary Tables S1 and S2 list the selected fields and their roles used in this paper.

Bank Marketing comes from a Portuguese bank’s real telemarketing calls, with 45,211 records and 17 attributes. We select age, marital_status, education, contact, duration, and campaign as quasi-identifiers, set job_categorical as the sensitive attribute, and use client_id only as an index for reconstruction. During preprocessing, we first remove records with severe missingness or obvious anomalies, then apply integer encoding to categorical fields and z-score normalization to numeric QIs to remove scale effects and stabilize autoencoder training. Categorical QIs remain discrete and are generalized according to their semantics.

Adult is derived from the 1994 U.S. Census, with 32,561 records and 15 fields in the original training set. For comparability with Bank Marketing, we construct a field set isomorphic to Supplementary Table S1 (Supplementary Table S2): we keep key attributes such as age, education, marital_status, occupation, and hours-per-week, and form the aligned fields via renaming/merging, where job_categorical remains the sensitive attribute. We remove records containing “?” first, apply label encoding to all categorical fields, and perform type validation plus z-score normalization on continuous attributes such as age, duration, and campaign, ensuring Adult and Bank Marketing match in both field count and type.

### 5.2. Experimental Settings

All experiments were conducted on 10,000 randomly sampled records from each dataset to ensure a fair comparison across the methods and to keep the runtime at a manageable level. The experiments run on Windows 11, with the implementations in Python 3.10. The hardware includes AMD Ryzen 5 5600 (six cores) and 32 GB RAM. The key hyperparameter settings for AE-LRHMA and baselines are listed in Table 1.

For the autoencoder setup, numeric QIs are z-score normalized. In the main configuration, categorical QIs are represented by integer codes and fed into the AE. To examine whether the representation learning stage is materially affected by categorical-QI encoding, we additionally evaluate a one-hot variant while keeping the remaining AE-LRHMA pipeline unchanged. The AE is a fully-connected encoder–decoder with ReLU activations; latent dimension *r* follows the setting listed in the corresponding latent dimension r is set as reported in Table 1. Training uses Adam (lr = 1 × 10^−3^), epoch = 50, batch = 128, MSE loss, and a fixed random seed (seed = 42).

To provide an initial evaluation under a multiple sensitive attribute setting, we additionally constructed a joint sensitive attribute on the Bank Marketing dataset by combining job_categorical and marital_status. All other components of the AE-LRHMA pipeline and the main hyperparameter configuration remained identical to those used in the primary Bank Marketing experiment.

### 5.3. Evaluation Metrics

To evaluate anonymization from multiple aspects—privacy strength, sensitive distribution balance, and computational efficiency—we adopt the following metrics on both datasets:

(1) Equivalence class (EC) size metrics. The number of equivalence classes, the average group size, and the maximum group size reflect the grouping granularity after anonymization. More ECs and a smaller average group size usually imply finer partitioning and potentially stronger privacy, but often with higher information loss; fewer ECs and overly large ECs may cause over-generalization and obscure a fine-grained structure. We report these three EC size metrics for all methods to compare their privacy–utility trade-offs [29].
(2) Sensitive distribution metrics. To characterize the concentration and diversity of the sensitive values within each EC, we use the maximum sensitive proportion $p_{max}\left(G\right)$ and sensitive entropy H(G) defined earlier (see Equations (2) and (3)). Here, $p_{max}\left(G\right)$ reflects whether a sensitive value is overly concentrated, while H(G) measures diversity. Since we care about overall trends, we report the average $p_{max}$ and average H across all ECs: a smaller $p_{max}$ indicates less concentration; a larger H indicates more diverse and balanced sensitive distributions.
(3) Runtime. We record the total runtime of one anonymization run under the same hardware and parameter settings. Since Bank Marketing and Adult are similar in scale and dimensionality, the runtime directly reflects overhead differences across hierarchical splitting and microaggregation.
(4) Information loss. We use the NCP (normalized certainty penalty)-based normalized information loss to quantify the overall precision loss. For numeric attributes, the NCP uses the ratio of the EC interval length to the global range; for categorical attributes, the NCP uses the ratio of the number of categories in an EC to the total number of categories [9,30]. The overall information loss is a weighted average across attributes; larger values indicate more severe loss. The “Information loss” column in Table 2 corresponds to this metric.

Based on these metrics, Table 2 summarizes the results of MDAV, APMCA, and AE-LRHMA on both datasets.

### 5.4. Visualization of AE-LRHMA Anonymization Results

To illustrate the anonymization behavior of AE-LRHMA in the learned latent space, we visualize the Adult and Bank Marketing datasets before and after anonymization. For each dataset, the following three subfigures are provided: the latent distribution before anonymization, the EC-based grouping produced by AE-LRHMA, and the convex hulls of several representative ECs in the latent space. For each dataset, the 2D subfigures use consistent coordinate ranges to facilitate a visual comparison.

**Figure 4:**
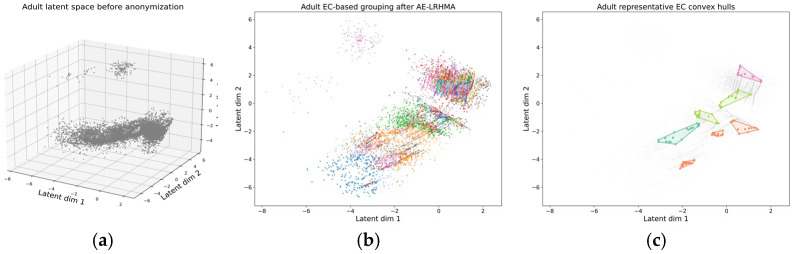
(**a**) Latent distribution before anonymization, (**b**) EC-based grouping after anonymization, (**c**) convex hulls of representative ECs.

**Figure 5:**
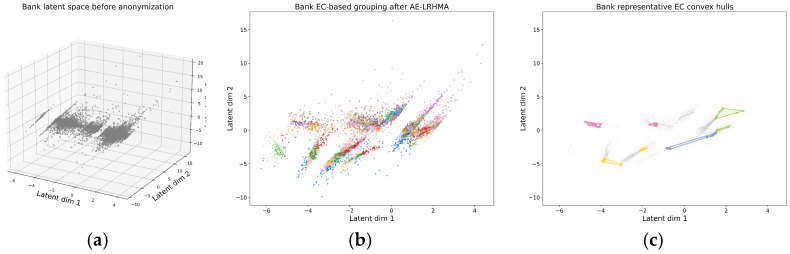
(**a**) Latent distribution before anonymization, (**b**) EC-based grouping after anonymization, (**c**) convex hulls of representative ECs.

As shown in Figure 4a and Figure 5a, before anonymization the latent representations exhibit irregular geometric structures, with visible local density differences, elongated regions, and a few isolated samples. This indicates that the learned latent space preserves the nontrivial structural characteristics of the original QI data.

Figure 4b and Figure 5b show the EC-based grouping results generated by AE-LRHMA in the latent space. Records assigned to different equivalence classes are displayed with different colors, making the partition structure more explicit. Compared with the original latent distribution, the anonymized results show a clearer EC organization while still preserving the main geometric arrangement of the data.

To further visualize the EC boundaries, Figure 4c and Figure 5c present the convex hulls of several representative ECs. These hulls provide an intuitive geometric view of the spatial extent of the ECs in the latent space. For clarity, only representative ECs are displayed in the hull visualization. Overall, the results indicate that AE-LRHMA forms interpretable EC partitions in the learned latent space while preserving the main structural characteristics of the data.

### 5.5. Comparison and Analysis of Three Methods

To quantitatively compare MDAV [3], APMCA [10], and AE-LRHMA, we report the EC size metrics, sensitive distribution metrics (average, average entropy H), runtime, and NCP-based information loss on both datasets (Table 2). The three methods show clear differences in grouping granularity, sensitive distribution balance, and computational cost.

For the Adult dataset, MDAV yields 1250 equivalence classes (ECs), with both the average and maximum group sizes equal to 8, indicating that most groups collapse to the k lower bound and the partition becomes highly fragmented. Although k-anonymity is satisfied, the resulting fine-grained intervals/category sets incur larger information loss, and the runtime is close to 200 s (the highest among the three). APMCA forms 411 ECs (average size ≈ 25; maximum size = 147), reflecting a tendency toward over-generalization; it achieves $p_{max}\;$= 0.3198, H = 2.4973, and NCP = 0.3861, improving over MDAV. AE-LRHMA produces 506 ECs (average size ≈ 20) with the maximum size capped within 24, leading to a more controlled group structure and the lowest information loss ($p_{max}\;$= 0.3124, H = 1.7690, NCP = 0.2808). In terms of runtime, AE-LRHMA (≈7.92 s) is comparable to APMCA (≈7.87 s) and substantially faster than MDAV. The lower entropy H of AE-LRHMA relative to APMCA reflects a utility-oriented trade-off in the sensitive value dispersion. Here, we set $h_{min}$ = 0 to disable the hard entropy gate, while still encouraging entropy-balanced splits via the entropy-improvement term in the split-scoring function (Equation (7)); the primary privacy control is enforced by $p_{max}$ together with group size constraints. The ERR/UMR linkage evaluation further suggests acceptable empirical disclosure risk under the configured thresholds.

Similar patterns are observed on the Bank Marketing dataset. MDAV produces a large number of small equivalence classes (1251 ECs; average/maximum size = 8) with NCP = 0.4516 and an approximate runtime of 197 s. APMCA forms 914 ECs (average size ≈ 11; maximum size = 22), resulting in a lower NCP (0.4087) and improved sensitive distribution indicators ($p_{max}\;$= 0.3792, H = 2.1501) compared with MDAV. AE-LRHMA forms 428 ECs (average size ≈ 23; maximum size constrained within 24) and achieves the lowest NCP (0.3373). Although its sensitive distribution indicators ($p_{max}\;$= 0.4303, H = 1.5064) are higher than those of APMCA, they remain within the configured thresholds, providing an interpretable privacy–utility compromise. In terms of runtime, AE-LRHMA (≈7.96 s) is faster than APMCA (≈12.91 s) and substantially faster than MDAV.

Overall, MDAV tends to compress ECs to the minimum size, causing fragmentation, higher information loss, and the longest runtime—serving as a conservative “privacy-first” baseline. APMCA significantly reduces runtime and information loss via aggregation, but EC sizes can become large in some cases, risking over-generalization. AE-LRHMA performs hierarchical partitioning in the latent space and combines local (*k*,*e*)-MDAV with sensitive distribution controls, achieving a more balanced trade-off across privacy, information loss, and efficiency, with the lowest NCP (0.2808/0.3373) and an acceptable runtime on both datasets—making it more suitable for practical tabular data anonymization.

### 5.6. Ablation Study

To further analyze the role of each component in AE-LRHMA, we construct three ablation variants on both datasets while keeping the other settings unchanged, as follows:

(1) **full-AE-LRHMA:** full model with autoencoder representation learning, latent space hierarchical splitting, and sensitive distribution constraints;
(2) **w/o-diversity:** keep AE and latent space partitioning but remove maximum sensitive proportion and entropy constraints, leaving only k-anonymity and EC size-related constraints;
(3) **w/o-AE:** remove AE representation learning and perform hierarchical splitting plus local microaggregation directly in the original QI space. The results are summarized in Table 3.

First, comparing full-AE-LRHMA with w/o-AE shows that the autoencoder representation learning module is crucial for stabilizing the EC structure and grouping granularity. Without AE, both datasets exhibit clear fragmentation: on the Adult dataset, the number of ECs increase from 506 to 1132 and the average group size drops from 20 to 9; on the Bank Marketing dataset, the number of ECs rise from 428 to 971 and the average size drops from 23 to 10, though the max group size is still capped by max_group_size (=24). This indicates that performing Mondrian-style recursive splits directly in the original high-dimensional QI space is more likely to cause axis-aligned “hard cuts” due to inconsistent scales, complex correlations, and sparse outliers, repeatedly generating small ECs near the k lower bound and destabilizing the overall grouping structure.

Although w/o-AE yields a lower NCP on both datasets, this decrease should not be interpreted as improved anonymization quality. It is mainly a degeneracy effect: smaller equivalence classes reduce the numerical width of interval generalization, which can lower the NCP while simultaneously fragmenting group structure and weakening risk controllability. By contrast, full-AE-LRHMA reconstructs and regularizes the high-dimensional quasi-identifier geometry in a compact latent space, so that split-dimension selection and median-based splitting better follow the underlying sample density. This reduces distance distortion and local noise, leading to more stable equivalence classes in both size and shape.

Second, with AE retained, comparing full-AE-LRHMA and w/o-diversity isolates the effect of sensitive distribution constraints. The two are almost identical in structural metrics (#ECs, average size, max size), indicating that, in the latent space, the EC geometry is mainly determined by AE + hierarchical splitting. However, after removing $p_{max}$ and entropy constraints, the average $p_{max}$ increases and the average entropy decreases, suggesting that, without explicit diversity control, local correlations between sensitive attributes and some QIs may still cause sensitive values to over-concentrate in certain ECs. With sensitive distribution constraints enabled, the CheckConstraints module can reject or further split candidate partitions with “excessive concentration or insufficient diversity”, suppressing extreme homogenization without significantly changing group size distributions.

Finally, in terms of efficiency, w/o-AE is fastest because it avoids AE training and latent mapping; w/o-diversity incurs slightly more overhead than the full model due to additional sensitive constraint checks and scoring computations. Overall, the added cost is acceptable and yields more stable EC structures and more controllable sensitive distributions. In summary, AE mainly stabilizes EC shapes and scales, while sensitive distribution constraints suppress worst-case sensitive concentration; the two complement each other in AE-LRHMA, enabling a more stable and interpretable trade-off among privacy strength, utility, and efficiency.

### 5.7. Representation Sensitivity to Categorical QI Encoding

Since categorical QIs enter the representation learning stage, their encoding may influence the geometry of the learned latent space and, consequently, the downstream partition structure. We therefore compare the main integer-coded configuration with a one-hot alternative while keeping the remaining AE-LRHMA pipeline unchanged.

Table 4 reports the comparison of the Adult and Bank Marketing datasets. The integer-coded setting corresponds to the main configuration used throughout this paper, whereas the one-hot setting changes only the encoding of the categorical QIs. The results show that the absolute values of the NCP, ERR, and UMR vary under one-hot encoding, indicating that categorical QI encoding affects the empirical operating point of the method. Nevertheless, AE-LRHMA continues to exhibit a meaningful privacy–utility trade-off under both representations. Overall, the main trade-off pattern remains qualitatively stable, suggesting that the method is sensitive to representation choice but not solely dependent on a particular categorical encoding.

### 5.8. Linkage-Attack Evaluation from the Attacker’s Perspective

To validate the empirical trend and stability of the containment linkage risk estimation (conservative/proxy upper-bound under stated assumptions) introduced earlier, we construct an external table T for both datasets and perform containment linkage under the setting that the QI set Q is known. For any external record *x* ∈ T, we compute its candidate EC set C(x) via Equation (8). Assuming no sensitive attribute prior knowledge, the attacker adopts the “most favorable candidate” strategy—guessing using the smallest EC in C(x) (Equation (9)). We then use Equation (11) as the expected proxy upper bound of identity-hit risk (ERR) and Equation (12) as the probability of being uniquely located into an EC (UMR). Importantly, the UMR measures the uniqueness of the EC localization, while the ERR measures the expected proxy upper bound of further identity hit under the most favorable candidate; therefore, they need not change in the same direction.

**Figure 6:**
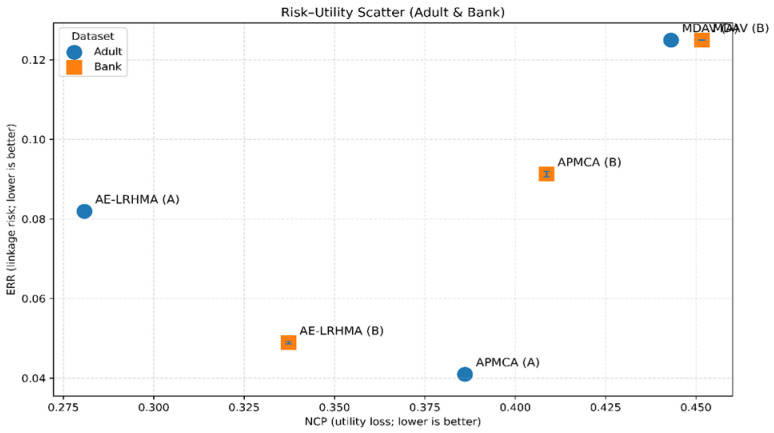
Risk–Utility Scatter (NCP–ERR) for the Adult and Bank Marketing datasets.

We vary the external sampling ratio ext_frac ∈ {0.05, 0.10, 0.20} and repeat the experiments with different random seeds. Table 5 and Table 6 report the ERR/UMR under different ext_frac and seeds. To visualize the overall “utility loss–linkage risk” trade-off, we further plot NCP vs. ERR as a risk–utility scatter plot (Figure 6), where each point corresponds to one experimental configuration; detailed tables are provided in Supplementary Tables S4 and S5.

From Table 5 and Table 6, the relative ranking across methods is generally stable under different ext_frac and random seeds, indicating the robustness of the attack evaluation. The overall trends are consistent with the EC structure and utility-loss analyses: MDAV often produces many small ECs near the k lower bound, making candidate sets easier to shrink and yielding a higher ERR; AE-LRHMA strikes a balance between the EC size and generalization strength, controlling risk at an acceptable level while achieving lower utility loss; APMCA’s risk behavior depends on its aggregation pattern and can vary across the datasets.

As shown in Figure 6, the risk–utility positions of different methods are clear: AE-LRHMA maintains a lower NCP on both datasets with moderate risk; APMCA has a lower risk on the Adult dataset but higher risk on the Bank Marketing dataset; MDAV lies in a region that is not advantageous in either risk or utility. Together, Table 5 and Table 6 and Figure 6 indicate that reporting only information loss or runtime is insufficient to reflect real privacy risk; attacker-perspective linkage evaluation complements the evidence chain and makes conclusions about the privacy–utility trade-off more complete.

### 5.9. Supplementary Evaluation with a Joint Sensitive Attribute

In real-world data publishing scenarios, multiple sensitive attributes may coexist. To examine whether AE-LRHMA remains effective under such conditions, we conducted an additional experiment on the Bank Marketing dataset using a joint sensitive attribute. Specifically, the attributes job_categorical and marital_status were combined into a single joint sensitive attribute, while the remaining anonymization workflow and hyperparameters were kept consistent with the original Bank Marketing experiment. Since marital_status became part of the sensitive attribute, the quasi-identifiers in this supplementary setting were age, education, contact, duration, and campaign.

**Figure 7:**
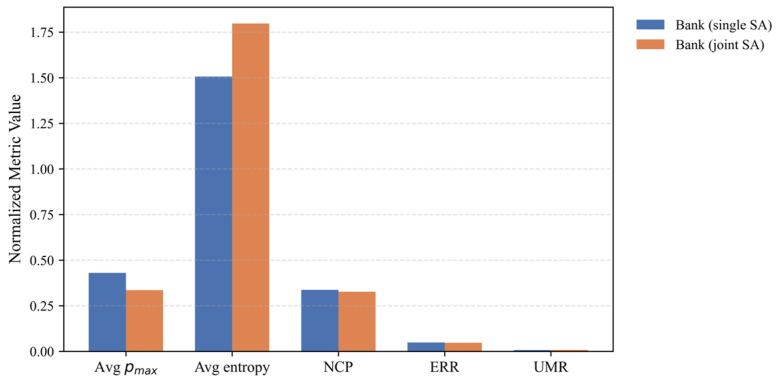
Comparison between the single SA and joint SA settings.

The results are summarized in Table 7 and further illustrated in Figure 7. Under the joint sensitive attribute setting, AE-LRHMA produced 648 equivalence classes with an average group size of 15.43 and a maximum group size of 24. Compared with the original single sensitive attribute configuration, the average sensitive concentration decreased to 0.3356 while the average entropy increased to 1.7970, indicating a more balanced distribution of sensitive information within the equivalence classes. At the same time, the utility-loss and attacker-oriented risk indicators remained close to the primary Bank Marketing experiment, with NCP = 0.3268, ERR = 0.0470, and UMR = 0.0080. These observations suggest that AE-LRHMA can still maintain a stable privacy–utility trade-off in an initial multiple sensitive attribute scenario.

## 6. Conclusions

This paper addresses the privacy leakage risks that arise when high-dimensional structured data are published and shared. Building on the classical hierarchical Mondrian anonymization framework, we introduce low-dimensional latent representations learned by an autoencoder and propose an autoencoder-enhanced hierarchical anonymization method, AE-LRHMA. The method first performs unsupervised representation learning over the quasi-identifiers to compress the original high-dimensional attributes into a more compact latent space. It then conducts Mondrian-style hierarchical partitioning in the latent space, integrates local (*k*,*e*)-MDAV microaggregation, and incorporates sensitive distribution constraints to explicitly control the equivalence class size as well as the concentration and entropy level of sensitive attributes within each equivalence class. Finally, it maps the partitioning results back to the original attribute space and applies interval/set generalization to produce the released data that satisfy the anonymization constraints.

Experiments on two public datasets, Bank Marketing and Adult, show that AE-LRHMA achieves a more balanced performance than classic MDAV and APMCA in terms of equivalence class granularity, sensitive attribute distribution balance, and runtime. On the one hand, it avoids the severe information loss caused by MDAV’s tendency to generate a large number of extremely small equivalence classes; on the other hand, it mitigates the over-generalization issue of APMCA, where equivalence classes can become overly large. Overall, AE-LRHMA can effectively strengthen group-level privacy protection while preserving data utility, demonstrating practical value and application potential for structured data anonymization in real-world scenarios. This finding is consistent with recent research trends in medical data anonymization, health data sharing, and privacy-enhancing machine learning, highlighting the practical importance of jointly considering privacy strength and data utility when designing anonymization algorithms [11,12,13,14,15]. An additional comparison with one-hot categorical-QI inputs shows that the empirical operating point of AE-LRHMA is influenced by the encoding used in the representation learning stage, while the overall privacy–utility trade-off remains qualitatively stable.

Nevertheless, this study has several limitations. First, AE-LRHMA remains within the equivalence class-based k-anonymity paradigm. The controls on maximum sensitive proportion, the optional entropy hard-gate threshold, and local sensitive dissimilarity are empirically motivated rather than formal privacy guarantees (e.g., differential privacy) and therefore do not provide strict theoretical bounds. Their privacy meaning is analyzed under the publishing–linkage-attack setting in Section 4, but their behavior under stronger threat models (e.g., interactive queries or active injection) still requires further investigation. Second, the current evaluation focuses on the Adult and Bank Marketing datasets. Although the main experiments use a single sensitive attribute, we additionally conducted a supplementary Bank Marketing experiment with a joint sensitive attribute, where AE-LRHMA still maintains a stable privacy–utility behavior in this initial multiple sensitive attribute setting. Nevertheless, broader evaluations on datasets with multiple sensitive attributes, highly imbalanced distributions, or temporal/tabular sequences remain necessary. Third, the current implementation is single-machine and sequential, and thus the experiments mainly serve controlled comparative evaluation rather than large-scale scalability testing. Future work will explore integrating principled randomization mechanisms to better connect with formal privacy frameworks and extending the method to multi-sensitive and temporal scenarios with improved scalability.

## Figures and Tables

**Table 1:** Key hyperparameter settings of AE-LRHMA.

Category	Symbol/Parameter	Meaning	Default
Privacy parameters	k	k-anonymity (minimum EC size)	8
	e	sensitive difference threshold for local (*k*,*e*)-MDAV	3
	$p_{max}$	upper bound of maximum sensitive proportion	0.8
	$h_{min}$	optional hard-gate entropy threshold for sensitive distribution	0.0
Split stopping	variance_threshold	stop splitting if the latent space variance is below this value	0.05
Group size cap	max_group_size	maximum equivalence class size	3k = 24
AE	r	latent dimension	3
	E	AE training epochs	50

**Table 2:** Performance comparison of three algorithms on Bank Marketing and Adult.

Dataset	Method	#ECs	Avg Group Size	Max Group Size	$\mathrm{Avg}.\;p_{max}$	Avg. Entropy H	Runtime	NCP
Adult	MDAV	1250	8	8	0.3447	2.2333	194.63 s	0.4431
Adult	APMCA	411	25	147	0.3198	2.4973	7.87 s	0.3861
Adult	AE-LRHMA	506	20	24	0.3124	1.7690	7.92 s	0.2808
Bank	MDAV	1251	8	8	0.3919	2.0392	197 s	0.4516
Bank	APMCA	914	11	22	0.3792	2.1501	12.91 s	0.4087
Bank	AE-LRHMA	428	23	24	0.4303	1.5064	7.96 s	0.3373

**Table 3:** Ablation results on Adult and Bank Marketing.

Dataset	Method	#ECs	Avg. Group Size	Max Group Size	$\mathrm{Avg}.\;p_{max}$	Avg. Entropy H	Runtime	NCP
Adult	full-AE-LRHMA	506	20	24	0.3124	1.7690	7.92 s	0.2808
Adult	w/o-diversity	520	20	24	0.3213	1.7431	7.12 s	0.2778
Adult	w/o-AE	1132	9	24	0.3842	1.4485	4.73 s	0.1340
Bank	full-AE-LRHMA	428	23	24	0.4303	1.5064	7.96 s	0.3373
Bank	w/o-diversity	442	23	24	0.4326	1.4887	6.567 s	0.3293
Bank	w/o-AE	971	10	24	0.4784	1.2525	3.52 s	0.3023

**Table 4:** Comparison of AE-LRHMA under integer-coded and one-hot categorical QI representations.

Dataset	Representation	NCP	ERR (ext_frac = 0.10)	UMR (ext_frac = 0.10)
Adult	Integer-coded	0.2808	0.0819 ± 0.0013	0.0054 ± 0.0011
Adult	One-hot	0.2176	0.0995 ± 0.0011	0.0138 ± 0.0035
Bank	Integer-coded	0.3373	0.0489 ± 0.0003	0.0076 ± 0.0036
Bank	One-hot	0.3262	0.1129 ± 0.0004	0.0072 ± 0.0036

Note: The integer-coded setting corresponds to the main AE-LRHMA configuration used throughout this paper. In the one-hot variant, only the representation of the categorical QIs is changed, while the remaining pipeline and parameter settings are kept unchanged. The ERR and UMR are reported at ext_frac = 0.10.

**Table 5:** Linkage attack risk on the Adult dataset.

Method	ext_frac = 0.05 (ERR/UMR)	ext_frac = 0.10 (ERR/UMR)	ext_frac = 0.20 (ERR/UMR)
APMCA	0.040748 ± 0.000777/0.9188 ± 0.0104	0.040948 ± 0.001193/0.9262 ± 0.0073	0.040968 ± 0.000418/0.9306 ± 0.0021
AE-LRHMA	0.082667 ± 0.002392/0.0032 ± 0.0023	0.081917 ± 0.001319/0.0054 ± 0.0011	0.082383 ± 0.001169/0.0050 ± 0.0019
MDAV	0.124950 ± 0.000112/0.0000 ± 0.0000	0.124950 ± 0.000068/0.0000 ± 0.0000	0.124913 ± 0.000034/0.0003 ± 0.0003

Note: The ERR and UMR are computed by Equations (11) and (12), respectively; when C(x) = ∅, the external record is considered unmatched and contributes 0 risk.

**Table 6:** Linkage attack risk on the Bank Marketing dataset.

Method	ext_frac = 0.05 (ERR/UMR)	ext_frac = 0.10 (ERR/UMR)	ext_frac = 0.20 (ERR/UMR)
APMCA	0.090957 ± 0.001188/0.9996 ± 0.0009	0.091268 ± 0.000715/0.9998 ± 0.0004	0.091432 ± 0.000320/ 0.9998 ± 0.0003
AE-LRHMA	0.049080 ± 0.000435/0.0080 ± 0.0035	0.048895 ± 0.000305/0.0076 ± 0.0036	0.049078 ± 0.000222/ 0.0082 ± 0.0021
MDAV	0.125000 ± 0.000000/0.0020 ± 0.0035	0.124975 ± 0.000056/0.0026 ± 0.0009	0.124913 ± 0.000056/ 0.0018 ± 0.0004

**Table 7:** Supplementary results under a joint sensitive attribute setting on the Bank Marketing dataset.

Setting	#ECs	Avg Group Size	Max Group Size	$\mathrm{Avg}.\;p_{max}$	Avg. Entropy H	Runtime	NCP	ERR	UMR
single SA	428	23	24	0.4303	1.5064	7.96	0.3373	0.0489	0.0076
joint SA	648	15.43	24	0.3356	1.7970	10.92	0.3268	0.0470	0.0080

## Data Availability

The Adult Census Income and Bank Marketing datasets analyzed in this study are publicly available from the UCI Machine Learning Repository. The implementation code is available at: https://github.com/sakula328/AE-LRHMA (accessed on 20 March 2026).

## Supplementary Materials

The following supporting information can be downloaded at: https://www.mdpi.com/article/10.3390/e28040372/s1, Table S1: Bank Marketing dataset: field configuration used in this paper; Table S2: Adult Census Income dataset: field configuration used in this paper; Table S3: Notations and symbols used in this paper; Table S4: Complete linkage-attack metrics on Adult (mean ± std over 5 random seeds); Table S5: Complete linkage-attack metrics on Bank Marketing (mean ± std over 5 random seeds); Table S6: Algorithm listing of AE-LRHMA (full pseudocode).
