# Supplementary material for: Autoencoder-Enhanced Hierarchical Mondrian Anonymization via Latent Representations
Source: Entropy (Basel). 2026 Mar 25;28(4):372. doi: 10.3390/e28040372 (PMC13114924; doi:10.3390/e28040372)
Supplement: Supplementary file 1 [file entropy-28-00372-s001.zip › entropy-4155348-supplementary.pdf]

entropy

MDPI

Supplementary Materials

Autoencoder-Enhanced Hierarchical Mondrian Anonymization via Latent Representations

Junpeng Hu [1,2], Tao Hu [2], Zhenwu Xu [1], Jinan Shen [2] and Minghui Zheng [1,2,*]

[1] School of Cyber Science and Engineering, Sichuan University, 610207 Chengdu, China
[2] College of Intelligent Science and Engineering, Hubei University for Nationalities, 445000 Enshi, China
* Correspondence: mhzheng3@163.com

Supplementary Materials: This document provides additional tables and algorithmic details to support the main manuscript, including Tables S1–S6.

Table S1: Bank Marketing dataset: field configuration used in this paper.

Field	Type	Role	Notes / characteristics
ID	Identifier	ID	Auto-incremented index; used only as the primary key
Age	Numeric	QI	Age (quasi-identifier)
Job_categorical	Categorical	SA	Job; mapped to integers and treated as the sensitive attribute
marital_status	Categorical	QI	Marital status; mapped to integers
education	Categorical	QI	Education level; category-to-integer encoding
contact	Categorical	QI	Contact type; used as a categorical QI
duration	Numeric	QI	Call duration; used as a numeric QI
campaign	Numeric	QI	Number of contacts; used as a numeric QI

Table S2: Adult Census Income dataset: field configuration used in this paper.

Field	Type	Role	Notes / characteristics
ID	Identifier	ID	Auto-incremented index; used only as the primary key
Age	Numeric	QI	Age (quasi-identifier)
Job_categorical	Categorical	SA	Job/occupation; mapped to integers and treated as the sensitive attribute
marital_status	Categorical	QI	Marital status; mapped to integers
education	Categorical	QI	Education level; category-to-integer encoding
contact	Categorical	QI	Relationship (renamed as contact)
duration	Numeric	QI	Hours-per-week (renamed as duration)
campaign	Numeric	QI	Capital-gain (renamed as campaign)

Note: To align with Bank Marketing, we keep the names contact/duration/campaign on Adult, corresponding to relationship / hours-per-week / capital-gain, respectively; the meanings and value definitions follow the original dataset.

Table S3: Notations and symbols used in this paper.

Symbol	Meaning	Remarks / Range
$D = \{(q_i,s_i) \mid i = 1,\cdots,n\}$	Original dataset	n: number of records
$q_i \in \mathbb{R}^d$	QI vector of the i-th record	d: QI dimensionality
s_i	Sensitive-attribute value of the i-th record	Single sensitive attribute setting
Q	Quasi-identifier set (QI set)	Used for linkage matching
S	Sensitive-attribute set (SA set)	Single SA in this paper
$G \subseteq D$	Equivalence class (EC)	
$\mathcal{G} = \{G_1,\cdots,G_m\}$	EC partition of dataset D	m: number of ECs
$P_G(v)$	Empirical distribution of sensitive value s in EC E	
$p_{\max}(G)$	Maximum sensitive proportion of EC E	
$H(G)$	Sensitive entropy of EC E	
k	Minimum EC size	
e	Sensitive-dissimilarity constraint for microaggregation	Used in the microaggregation stage
$p_{\max}$	Upper bound on maximum sensitive proportion	
$h_{\min}$	Entropy lower-bound threshold	
$(k,e,p_{\max},h_{\min})$-匿名	Definition of the hybrid privacy constraint	Defined in Section 2.1
$X_Q \in \mathbb{R}^{n\times d}$	Preprocessed QI feature matrix	Each row corresponds to one record
$f_\theta(\cdot)$	Encoder	Parameters
$g_\phi(\cdot)$	Decoder	Parameters
$\mathcal{L}(\theta,\phi)$	Mean reconstruction loss of the autoencoder	L2 reconstruction loss is used
r	Latent dimension	
E	Number of training epochs for the autoencoder (AE)	Default value
$Z = f_\theta(X_Q)$	Latent representation matrix	
z_i	Latent representation of the i-th record	Used for splitting/distance computation
G_L,G_R	Left/right subsets after splitting the current node	Binary split result
Δ_{span}	Change of latent-space span before/after splitting	Composite scoring term
Δ_H	Entropy-gain term induced by a split	Composite scoring term
$\alpha \in [0,1]$	Weight coefficients in the composite scoring function	
$Score(G \to G_L,G_R)$	Composite split-scoring function	Example form is given in Section 2.2
σ^2	Variance of latent representations at the current node	Algorithm 1
τ_{var}	Variance threshold (variance_threshold)	Algorithm 1
τ_H	Entropy threshold (entropy_min)	Algorithm 1
max_group_size	Upper bound on EC size (max_group_size)	See Table S3
δ	SensitiveDiff (sensitive dissimilarity)	Algorithm 1
W	Work queue of subsets to be processed	Algorithm 1

https://doi.org/10.3390/1e28040372

C	Candidate subset set (for microaggregation)	Algorithm 1
$\mathcal{R}$	Remainder set / records to be assigned	Algorithm 1
CheckConstraints(·)	Constraint validation function	
LocalLRKEMDAV(·)	Local microaggregation subroutine	Based on (k, e)-MDAV
AssignRemainder(·)	Remainder assignment	Merged into a valid EC by composite distance
GeneralizeInOriginalSpace(·)	Generalization output in the original space	Numeric interval / categorical set
NCP	Normalized Certainty Penalty (NCP)	Larger values indicate higher information loss
Symbol	Meaning	Remarks / Range
T	External table	Used in linkage attacks
$x \in T$	External-table record	Matched to the released table via QIs
EC_j	j-th equivalence class (EC)	Elements in the attack candidate set
$C(x)$	Candidate EC set for an external record x	Containment linkage
$P_r(hit\|x)$	Upper bound on EC-hit probability	
m(x)	Minimum EC size among candidates for x	Section 2.3 and Supplementary tables
ERR	Expected Re-identification Risk (ERR)	Lower is safer
UMR	Unique Match Rate (UMR)	
ext_frac	Sampling ratio of the external table (ext_frac)	0.05 / 0.10 / 0.20
seed	Random seed	0–4
mean±std	Aggregation over multiple seeds	Section 3.7 and Supplementary tables
match_rate	Match rate (reported in Supplementary tables)	Supplementary Tables S2–S3
avg_candidate_ec	Average number of candidate ECs (Supplementary)	Supplementary Tables S2–S3

Table S4: Complete linkage-attack metrics on Adult (mean ± std over 5 random seeds).

Method	ext_frac = 0.05	ext_frac = 0.10	ext_frac = 0.20
AE-LRHMA	ERR0.082667±0.002392<br>UMR0.0032±0.0023<br>match_rate1.0000±0.0000<br>avg_candidate_ec47.492±1.126<br>avg_min_group_size16.128±0.459	ERR 0.081917±0.001319<br>UMR 0.0054±0.0011<br>match_rate1.0000±0.0000<br>avg_candidate_ec47.360±0.605<br>avg_min_group_size16.272±0.253	ERR 0.082383±0.001169<br>UMR 0.0050±0.0019<br>match_rate1.0000±0.0000<br>avg_candidate_ec47.833±0.332<br>avg_min_group_size16.182±0.224
APMCA	ERR 0.040740±0.000777<br>UMR 0.9188±0.0104<br>match_rate1.0000±0.0000<br>avg_candidate_ec1.082±0.010<br>avg_min_group_size51.497±1.051	ERR 0.040948±0.001193<br>UMR 0.9262±0.0073<br>match_rate1.0000±0.0000<br>avg_candidate_ec1.075±0.007<br>avg_min_group_size 50.978±0.990	ERR 0.040968±0.000418<br>UMR 0.9306±0.0021<br>match_rate1.0000±0.0000<br>avg_candidate_ec1.070±0.002<br>avg_min_group_size51.483±0.616
MDAV	ERR 0.124950±0.000112<br>UMR 0.0000±0.0000<br>match_rate0.9996±0.0009<br>avg_candidate_ec508.244±15.563	ERR 0.124950±0.000068<br>UMR 0.0000±0.0000<br>match_rate0.9996±0.0005<br>avg_candidate_ec508.110±9.159	ERR 0.124913±0.000034<br>UMR 0.0003±0.0003<br>match_rate0.9993±0.0003<br>avg_candidate_ec513.375±4.165

https://doi.org/10.3390/1e28040372

	avg_min_group_size8.000±0.000	avg_min_group_size8.000±0.000	avg_min_group_size 8.000±0.000

Table S5: Complete linkage-attack metrics on Bank Marketing (mean ± std over 5 random seeds).

Method	ext_frac = 0.05	ext_frac = 0.10	ext_frac = 0.20
AE-LRHMA	ERR0.049080±0.000435<br>UMR0.0080±0.0035<br>match_rate1.0000±0.0000<br>avg_candidate_ec41.855±1.265<br>avg_min_group_size21.360±0.129	ERR0.048895±0.000305<br>UMR0.0076±0.0036<br>match_rate1.0000±0.0000<br>avg_candidate_ec41.799±0.681<br>avg_min_group_size21.414±0.083	ERR0.049078±0.000222<br>UMR0.0082±0.0021<br>match_rate1.0000±0.0000<br>avg_candidate_ec41.483±0.580<br>avg_min_group_size 21.368±0.061
APMCA	ERR0.090957±0.001188<br>UMR0.9996±0.0009<br>match_rate1.0000±0.0000<br>avg_candidate_ec1.000±0.001<br>avg_min_group_size11.710±0.131	ERR0.091268±0.000715<br>UMR0.9998±0.0004<br>match_rate1.0000±0.0000<br>avg_candidate_ec1.000±0.000<br>avg_min_group_size11.657±0.099	ERR0.091432±0.000320<br>UMR0.9998±0.0003<br>match_rate1.0000±0.0000<br>avg_candidate_ec1.000±0.000<br>avg_min_group_size 11.642±0.043
MDAV	ERR0.125000±0.000000<br>UMR0.0020±0.0035<br>match_rate1.0000±0.0000<br>avg_candidate_ec567.104±15.966<br>avg_min_group_size8.000±0.000	ERR0.124975±0.000056<br>UMR0.0026±0.0009<br>match_rate0.9998±0.0004<br>avg_candidate_ec565.377±6.270<br>avg_min_group_size8.000±0.000	ERR0.124912±0.000056<br>UMR0.0018±0.0004<br>match_rate0.9993±0.0004<br>avg_candidate_ec563.893±8.000<br>avg_min_group_size8.000±0.000

Table S6: Algorithm listing of AE-LRHMA (full pseudocode).

Algorithm 1. AE-LRHMA

Input: Dataset$D$; QI set$Q$; sensitive attribute S
Privacy parameters k,e,$p_{\max}$,$h_{\min}$
AE parameters r,E; thresholds τ_{var}(variance_threshold),τ_H(entropy_min)
Output: Anonymized dataset $\tilde{D}$

X_Q ←PreprocessQI(D,Q)
(f_θ,g_ϕ) ←TrainAE(X_Q,r,E)
Z ←$f_\theta(X_Q)$
W ← {{all record indices}};C ← $\emptyset$; $\mathcal{R}$←$\emptyset$
while $W \neq \emptyset$ do
G ← Pop(W);n ← $|G|$
δ ←SensitiveDiff(G,S)
if $k \leq n \leq 2k$ and $\delta \leq e$ then
C ←C ∪ {G}
else if $n > 2k$ then
σ^2 ←Variance(Z_G)
if $\sigma^2 > \tau_{var}$ then
a ← SelectSplitDim(Z_G)
m ← Median(Z_G[:,a])
(G_L,G_R) ←Split(G,Z_G,a,m)
if CheckConstraints (G_L,G_R,k,e,$p_{\max}$,$h_{\min}$,τ_H)then
W ←W ∪ {G_L,G_R}
else $\mathcal{R}$ ←$\mathcal{R}$ ∪ {G}
else $\mathcal{R}$ ←$\mathcal{R}$ ∪ {G}
else $\mathcal{R}$ ←$\mathcal{R}$ ∪ {G}
end while
$\mathcal{G}$ ← $\emptyset$

https://doi.org/10.3390/1e28040372

for each $G \in C$ do
(groups,rem) ←LocalLRKEMDAV(G, Z, k,e,p_{max},h_{min},S)
$\mathcal{G}$ ←$\mathcal{G}$ ∪ groups
$\mathcal{R}$ ←$\mathcal{R}$ ∪ rem
end for
AssignRemainder($\mathcal{R}$, $\mathcal{G}$,Z)
$\tilde{D}$ ←GeneralizeInOriginalSpace($\mathcal{G}$,D,Q)

https://doi.org/10.3390/1e28040372
